# Use and Effectiveness of Quitlines for Smokers With Diabetes: Cessation and Weight Outcomes, Washington State Tobacco Quit Line, 2008

**DOI:** 10.5888/pcd10.120324

**Published:** 2013-06-27

**Authors:** Gillian L. Schauer, Terry Bush, Barbara Cerutti, Lisa Mahoney, Juliet R. Thompson, Susan M. Zbikowski

**Affiliations:** Author Affiliations: Terry Bush, Lisa Mahoney, Susan M. Zbikowski, Alere Wellbeing, Clinical and Behavioral Sciences, Seattle, Washington; Barbara Cerutti, Imperial College London and Imperial College Healthcare National Health Service Trust, London, UK; Juliet R. Thompson, Washington State Department of Health, Tobacco Prevention and Control Program, Tumwater, Washington.

## Abstract

**Introduction:**

Having diabetes and smoking increases the risk of morbidity and mortality. However, cessation-related weight gain, a common side effect during quitting, can further complicate diabetes. Evidence-based telephone quitlines can support quitting but have not been studied adequately in populations with chronic diseases such as diabetes. The purpose of this study was to evaluate the use and effectiveness of a tobacco quitline among tobacco users with diabetes. Cessation-related weight concerns and weight gain were also assessed.

**Methods:**

We administered a telephone-based follow-up survey to tobacco users with and without diabetes 7 months after their enrollment in a quitline. We collected and analyzed data on demographics, tobacco use, dieting, weight concern, quitting success (7- and 30-day point prevalence), and weight gain. We computed summary statistics for descriptive data, χ^2^ and *t* tests for bivariate comparisons, and multivariable analyses to determine correlates of cessation.

**Results:**

Tobacco users with diabetes used the quitline in a greater proportion than they were represented in the general population. Quit rates for those with and without diabetes did not differ significantly (24.3% vs 22.5%). No significant differences existed between groups for weight gain at follow-up, regardless of quit status. However, participants with diabetes reported more weight gain in previous quit attempts (34.2% vs 22.4% gained >20 lbs, *P* = .03). Weight concern was a significant correlate of continued smoking, regardless of diabetes status.

**Conclusions:**

Results suggest that quitlines are effective for participants with diabetes, but tailored interventions that address weight concerns during cessation are needed.

## Introduction

People with diabetes smoke at the same rate as people in the general population, despite excess health morbidity and mortality (1,2). In people with diabetes, cigarette smoking increases the risk of macrovascular complications, including circulatory, cardiovascular, and coronary heart disease (3,4), and microvascular complications, including kidney disease (5). Quitting smoking is essential to reduce the onset and exacerbation of diabetes (6).

However, weight gain is a potential barrier to successful quitting. It is common for people to gain 10 to 15 pounds after quitting (7,8) and those who are heavy smokers or are already overweight can gain considerably more (7,8). People with diabetes are more likely than those without diabetes to be overweight or obese (9), so excessive weight gain during cessation can present a substantial health hazard (10). In addition to actual weight gain, concern about gaining weight after quitting is common (11,12), and both hinder cessation (13,14). Although weight gain and weight concern are important factors in the initiation and continuation of smoking, few data exist on the effect of weight-related issues on cessation in tobacco users with diabetes.

Numerous evidence-based resources exist to support successful quitting, including pharmacotherapy and counseling provided through state tobacco quitlines (15). Toll-free, telephone-based tobacco quitlines are 1 of the most cost-effective treatment resources, yet even among the general population, they typically reach only 1% to 3% of smokers nationwide (16). Formative research suggests that people with diabetes have low awareness and use of effective cessation treatments including medication and quitlines (17). Although most cessation research has focused on the general population or related disease groups such as cardiovascular or lung disease (4), no published research has evaluated the use of a tobacco cessation quitline in a population of smokers with diabetes.

The primary purpose of this study was to describe the reach and effectiveness of a tobacco quitline among a sample of people with diabetes compared with those without the disease. A secondary aim was to assess the impact that concerns about weight gain have on quitting success.

## Methods

### Sample

Participants for this study were tobacco users aged 18 or older who called the Washington State Tobacco Quit Line (the quitline) from May through September 2008. For this cross-sectional study, we surveyed a census of tobacco users who reported a diagnosis of diabetes at the time of quitline registration and met other eligibility criteria and compared them with a matched group who registered for quitline services during the same period but did not report having diabetes. To control for differences that might affect quit rates (the dependent variable), we matched participants by sex, insurance status, and smoking dependence. Only 1 participant per household was included in the study; pregnant women were excluded because of the possibility of a pregnancy-related diabetes diagnosis.

At the time of data collection, the quitline offered free cessation services to any state resident aged 18 or older, including an initial counseling call of up to 30 minutes, self-management materials mailed to the caller, provision of nicotine patch or gum medication (if indicated), and referral to community-based cessation resources. The number of follow-up counseling calls (1 call vs up to 5 calls) and the amount of medication (2 vs 8 weeks) varied by population; uninsured, Medicaid-insured, and those referred from Veterans Affairs and Indian Health Services received the most intensive treatment options. Data on the Medicaid-insured population were available only for May and June because the state began offering separate Medicaid-only quitline services in July 2008. However, the separate Medicaid-only quitline services were available only for Medicaid fee-for-service participants. Other Medicaid participants may have been eligible for quitline services (and thus study recruitment) during the study period. This study was conducted by Alere Wellbeing in collaboration with the Washington State Department of Health with approval from the Western Institutional Review Board.

Data came from 1) information collected via telephone at the time of quitline registration, 2) a 7-month follow-up telephone survey, 3) automated process data collected at Alere Wellbeing (eg, number of counseling calls completed) and 4) comparison data from the 2008 Washington State Behavioral Risk Factor Surveillance System (BRFSS) survey. The 7-month follow-up telephone survey was administered from December 2008 through April 2009 by trained survey staff. To increase the survey response rate, a prenotification letter was mailed to participants about 10 days before survey administration. Participants were also offered a $20 gift card for completing the survey. If the interviewer could not reach a participant after 11 attempts, the survey was considered unanswered.

### Measures and definition of concepts

Quitline registration data included participant demographics, tobacco use and cessation history, stage of readiness to quit, and prior use of pharmacotherapy. Chronic disease status was assessed by asking “Have you been diagnosed with any of the following chronic conditions: Asthma? Chronic obstructive pulmonary disease or emphysema? Diabetes? Heart disease?” Diabetes status was further clarified by asking participants if they had ever been told by a doctor that they had diabetes, a core question from the Centers for Disease Control and Prevention’s (CDC’s) 2007 Behavioral Risk Factor Surveillance System Survey (http://www.cdc.gov/brfss/). Participants who were prediabetic or were diagnosed with diabetes only during pregnancy were excluded from analyses.

Seven-day and 30-day tobacco point-prevalence quit rates were based on a respondent’s self-report of being tobacco-free for the last 7 days or more, or 30 days or more at the time of the 7-month survey (18). Abstinence rates were computed by using both the responder and intent-to-treat methodology. Among continued smokers, intention to quit and reduction in amount of cigarettes smoked were also assessed.

Quitline use among tobacco users with diabetes was measured by a “reach effect ratio” based on the proportion of smokers aged ≥18) with diabetes who enrolled in quitline services in 2008 divided by the proportion of smokers with diabetes in the state (19). A reach ratio of 1.0 indicates that the quitline reaches the subgroup proportionally to its distribution in the smoking population in that state; ratios less than 1 indicate lower reach and greater than 1 indicate higher reach.

Self-reported height and weight, physical activity, dieting, level of concern about gaining weight, perceived risk for relapse if they were to gain weight, prior weight gain due to quitting, perceived weight and change in weight, and postcessation weight change (among those who quit) were also measured.

Self-reported depression was assessed by using the Patient Health Questionnaire-2 (20): “Over the last 2 weeks how often have you been bothered by the following problems? 1- Having little interest or pleasure in doing things? 2- Feeling down, depressed or hopeless?” A mean score on the 2 items was computed, with the recommended cut point of 3 or more describing clinically significant depression (20). The mean score on 1 item was used to determine self-reported anxiety symptoms: “Over the last 2 weeks how often have you been bothered by: feeling nervous, anxious, or on the edge?” Panic was assessed with a yes/no question: “During the past 2 weeks did you have any episodes of panic or fear?”

### Statistical analysis

Summary statistics were computed for descriptive data. Those who completed the survey were compared with those who did not to assess differences in individual characteristics. Chi-square and *t* tests were used for bivariate comparisons between groups. We conducted multiple logistic regression analyses by using SAS version 9.2 (SAS Institute Inc, Cary, North Carolina) to test for independent associations between the 2 groups in quit rates after controlling for demographic and tobacco use characteristics. Correlates of quitting were identified for the total sample and for the subsample with diabetes. Despite matching, sex was included in the multivariable analyses to control for possible selection bias (21), since both diabetes status (the key independent variable) and tobacco abstinence (the outcome variable) have been found to differ by sex (22). Less than 10% of responses were “do not know” or missing. These were omitted from the analyses. Significance was set at ɑ =.05 for all analyses.

## Results

### Quitline use

According to the weighted prevalence rates from the 2008 BRFSS in Washington State, 15.7% (95% confidence interval [CI] = 15.0–16.4) of residents aged 18 or older smoked. No statistical difference in diabetes prevalence was found between adults who smoked and the general adult population in Washington (6.1% vs 6.0%, excluding those diagnosed only during pregnancy). Quitline registrations from 2008 showed that 8.3% (n = 1,077) of all adults who registered for quitline services reported a diagnosis of diabetes. This represents a quitline reach effect ratio of 1.36 (8.3%/6.1%), indicating that in Washington State, smokers with diabetes used the quitline in a higher proportion than they were represented in the general population of smokers.

### Sample characteristics

Six hundred eligible tobacco users who registered for services with the quitline between May 1, 2008, and September 30, 2008, were eligible to participate in the 7-month survey (261 participants with diabetes and 339 participants without diabetes) (Figure). After launching the survey, we learned that approximately 19% of identified participants had either disconnected or wrong telephone numbers. The survey response rate was 40.3%, yielding a final sample of 242 participants (111 with diabetes, 131 without diabetes). Few differences existed between respondents and nonrespondents. Participants with diabetes were just as likely to complete the 7-month survey as those without diabetes (*P* = .34). Compared to nonrespondents, survey respondents were older (47.5 years vs 42.6 years; *P* < .001), less likely to be uninsured (10.7% vs 20.4%; *P* < .003), and more likely to have smoked for 20 years or more (75.6% vs 64.5%, *P* < .01).

**Figure:**
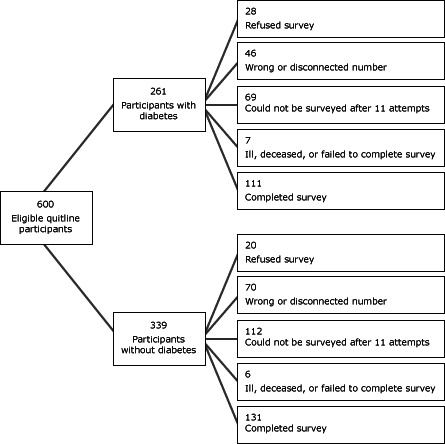
Recruitment process for survey of eligible tobacco users who sought help from the Washington State Quit Line, May 2008–September 30, 2008.

Respondents with diabetes were similar to those without diabetes in sex, race/ethnicity, education, insurance status, and current level of anxiety or panic (Table 1). Those with diabetes were older and more likely to report having significant depressive symptoms. Of the smokers with diabetes, 13% reported a diagnosis of type 1 diabetes, 78.9% reported a diagnosis of type 2 diabetes, and 8.2% reported they did not know. Both groups reported that the primary reason for calling the quitline was their desire or need to quit smoking, followed by advice to quit by family or health professionals.

**Table 1:** Demographics and Tobacco Use and Weight-Related Characteristics Among Quitline Participants With and Without Diabetes, Washington State, 2008

Characteristic	Diabetes			No Diabetes		
	n	%	Mean (SD)	n	%	Mean (SD)
**Sex, n (%)**						
Female	111	67.6	NA	131	67.9	NA
**Age^a^, y**						
18-25	111	1.8	51.0 (11.07)	131	12.2	44.6 (13.6)
26-40		13.5			27.5	
41-60		69.4			45.8	
>60		15.3			14.5	
**Race/ethnicity**						
Hispanic	109	2.7	NA	128	4.7	NA
Non-Hispanic		97.3			95.3	
White	110	79.1		129	86.0	
Nonwhite		20.9			14.0	
**Education**						
High school graduate or less	106	50.0	NA	129	52.7	NA
More than high school graduate		50.0			47.3	
**Mental health status^b^**						
Depression scale (range, 0-6)	108	45.4	2.4 (1.9)	127	30.2^b^	1.8 (2.0)^b^
Depressive symptoms (score >3)						
Anxiety scale		30.6	1.1 (1.1)		29.9	1.0 (1.1)
Percentage with panic (yes/no scale)						
**Other chronic diseases**						
Asthma	111	32.4	NA	131	19.0^b^	NA
Chronic obstructive pulmonary disease		30.6			19.9^b^	
Heart disease		15.3			11.5	
>1 of the above		8.7			10.7^b^	
**Tobacco type reported at enrollment**						
Cigarettes	108	96.3	NA	128	97.7	NA
Other tobacco type		3.7			2.3	
**Smoking level (no. of cigarettes per day)**						
Light (1-14)	111	23.4	NA	131	29.0	NA
Moderate (15-20)		41.4			34.3	
Heavy (≥21)		35.2			36.7	
**No. of years used tobacco**						
<20	94	13.8	NA	103	34.0	NA
≥20		86.2			66.0	
**Time to first cigarette**						
≤5 min after waking	109	54.1	NA	129	52.7	NA
**Use of nicotine replacement therapy**						
Yes	111	67.6	NA	131	75.6	NA
**Body mass index (BMI)^c^**						
Obese (BMI ≥30)	102	56.9	33.5 (9.5)	121	35.8	28.4 (7.7)
Overweight (BMI 25.0-29.9)		30.4			267.6	
Normal weight or underweight (BMI 18.0-24.9)		12.7			36.6	
**Perceived body weight**						
Very overweight	108	29.6	NA	126	12.7^a^	NA
Overweight		50.0			43.6	
Normal weight or underweight		20.4			43.7	
**Dieting status**						
Not currently dieting	99	50.5	NA	120	60.9	NA
Currently dieting to lose weight		27.3			18.3	
Currently dieting to keep weight as it is		22.2			20.8	
**Physical activity level**						
Days per week of moderate physical activity	101	NA	3.23 (2.87)	121	NA	3.90 (2.48)
**Concerns about weight gain after quitting^d^**						
Scored ≥5 on a 1 to 10 Likert scale^e^	83	62.6	NA	97	52.6	NA
**Likelihood of relapse due to weight gain**						
Scored ≥5 on a 1 to 10 Likert scale^e^	102	28.4	NA	115	29.6	NA

Abbreviations: SD, standard deviation; NA, not available.

a *P* < .001.

b *P* < .05.

c BMI was calculated as weight divided by height squared multiplied by 703 ([lb/in^2^] × 703).

d Among those who continued to smoke.

e Where 1 = not concerned, and 10 = very concerned.

Participants with diabetes did not differ from those without diabetes on the type of counseling call program (one call vs multiple calls); 41% of participants with diabetes and 45% of those without the disease enrolled in the multiple proactive counseling call program (up to 5 proactive counseling calls). No significant difference was found between groups in their satisfaction with quitline services or with their use of or satisfaction with quitline-provided nicotine replacement therapy (NRT). Approximately 68% of quitline users with diabetes used NRT compared with about 76% quitline users without diabetes.

### Tobacco use characteristics

Most participants reported smoking cigarettes rather than other forms of tobacco (Table 1). Most were heavy, established smokers and more than half reported having their first cigarette of the day within 5 minutes of waking. Many also reported having other chronic diseases such as asthma, chronic obstructive pulmonary disease, or heart disease. Participants with diabetes reported smoking for significantly more years than those in the comparison group (86.2% vs 66.0% reported ≥20 years of smoking, *P* < .001) (Table 1).

### Weight concern characteristics

Compared with participants without diabetes, participants with the disease were significantly more likely to be obese (56.9% vs 35.8% with BMI >30, *P* < .001) and to rate themselves as being very overweight (29.6% vs 12.7%, *P* < .001) (Table 1). Participants with diabetes did not differ from those without diabetes in dieting behaviors or physical activity levels. Although no differences in weight concern or perceived risk of relapse due to weight gain after quitting existed between participants with and without diabetes, nearly two-thirds of participants with diabetes were worried about possible weight gain, although less than one-third thought they would return to smoking if they gained excessive weight after cessation.

### Tobacco cessation outcomes

In bivariate analyses, participants with diabetes reported quit rates that were similar to those of participants without diabetes (24.3% vs 22.5%, 30-day respondent quit rate; *P* = .73) (Table 2). A separate analysis that further segmented those without diabetes by other chronic disease status (with vs without other chronic disease) found no significant difference in quit rates (diabetes group, 24.3%; group with other chronic disease, 26.5%; group without chronic disease, 20.0%; *P* = .84).

**Table 2:** Unadjusted Quit Rates and Weight Changes Among Participants With and Without Diabetes, Washington State, 2008

Unadjusted Quit Rates	Diabetes		No Diabetes	
Respondent 7-day quit rate	n	% or mean (SD)	n	% or mean (SD)
Quit	111	28.8	129	26.4
**Intent-to-treat 7-day quit rate**				
Quit	261	12.3	339	10.0
**Respondent 30-day quit rate**				
Quit	111	24.3	129	22.5
**Intent-to-treat 30-day quit rate**				
Quit	261	10.3	339	8.5
**Weight gain in prior quit attempts^a^**				
Mean (SD)	16.9 (24.21)		10.29 (18.0)^b^	
Gained <20 lbs	80	65.8	99	77.8
Gained ≥20 lbs		34.2		22.2^c^
**Weight change since calling the quitline**				
No change in weight	108	41.7	126	45.2
Gained weight		34.3		32.6
Lost weight		24.1		22.2
Weight gained, lbs, mean (SD)	19.2 (15.9)		15.4 (9.7)	
Range, lbs	3–75		5–40	
**Weight change among quitters**				
No change in weight	31	29.0	33	36.4
Gained weight		51.6		36.4
Lost weight		19.3		27.3
Weight gained, lbs, mean (SD)	23.2 (22.1)		14.7 (8.0)	

a Among those who made a prior quit attempt.

b *P* < .05.

However, participants with diabetes reported more weight gain in prior quit attempts than those without diabetes (34.2% vs 22.2% reported gaining >20 lbs; *P* < .05). Participants with diabetes who were successful in quitting smoking through the quitline were more likely to report weight gain than those who were successful and did not have diabetes, although results were not significant (*P* = .43). Amount of weight gained was also higher but not significantly so (23.2 lbs vs 14.7 lbs; *P* = .20) (Table 2).

Consistent with the bivariate results, multivariable results revealed that participants with diabetes had similar odds of reporting tobacco abstinence at either 7 or 30 days compared with those without diabetes (OR, 0.8; 95% CI, 0.38–1.55 and OR, 0.7, 95% CI, 0.36–1.55, respectively) after adjusting for BMI, age, sex, NRT use, weight concern, and mean depression score (Table 3).

**Table 3:** Correlates of Tobacco Cessation Among the Total Sample With and Without Diabetes, Washington State, 2008

Selected Characteristic	Diabetes	**No Diabetes**
	7-Day Tobacco Abstinence, OR (95% CI)	30-Day Tobacco Abstinence, OR (95% CI)
Diabetes status (diabetes vs no diabetes)	0.8 (0.38–1.55)	0.7 (0.36–1.55)
BMI^a^ (normal vs overweight or obese)	1.5 (0.97–2.25)	1.3 (0.84–2.00)
Age	1.0 (0.98–1.04)	1.0 (0.98–1.04)
Sex (female vs male)	2.3 (1.09–4.82)	2.0 (0.94–4.43)
NRT use	1.4 (0.63–2.93)	1.3 (0.57–2.86)
Weight concern (≥5 vs <5, on a 10 point scale)	0.8 (0.65–0.88)	0.8 (0.67–0.91)
PHQ-2 Depression score (<3 vs >3)^b^	1.1 (0.56–2.26)	0.9 (0.44–1.84)

Abbreviations: OR, odds ratio; CI, confidence interval; BMI, body mass index; NRT, nicotine replacement therapy.

a BMI was calculated as weight divided by height squared multiplied by 703 (lb/in^2^ × 703).

b Response options to the 2 PHQ-2 items are on a 0-3 severity scale. We computed the mean score (0 minimum possible, 6 maximum possible).

The only correlate of 7-day and 30-day tobacco abstinence among the total sample was weight concern; those who expressed greater perceived risk of relapse due to weight gain were less likely to report abstinence (7-day OR, 0.8; 95% CI, 0.65–0.88; 30-day OR, 0.8; 95% CI, 0.67–0.91). However, when analysis was restricted to the subsample with diabetes, weight concern was not a significant predictor of quitting (OR, 0.8; 95% CI, 0.66–1.03; data not shown).

## Discussion

Although tobacco quitline use is low among the general population (16), results from this study indicate that the proportion of tobacco users with diabetes who used the quitline was greater than their representation in the general statewide population. This finding is important, given that diabetes prevalence is increasing nationally and smoking is a behavioral risk factor that contributes both to causing and complicating the disease (23). Higher proportional use of the quitline among those with diabetes as compared with the general population may be the result of a concerted effort by the Washington State Department of Health to integrate cessation treatment referrals into state chronic disease systems (24). Another factor may be that diagnosis of a chronic condition, especially one related to smoking (eg, diabetes), has been shown to create an impetus to quit smoking (25).

In addition to use, a major finding of this study is that having diabetes did not affect quit rates, even after adjusting for individual differences such as age, sex, weight concern, depression, use of NRT, and BMI. Furthermore, participants with diabetes had similar odds of reporting tobacco abstinence compared with those without diabetes after adjusting for the existence of other chronic diseases, supporting evidence that chronic disease does not hinder successful cessation (25).

Although participants with diabetes were older, had smoked longer, and were more likely to be depressed than those without diabetes, these factors did not decrease their likelihood of quitting. This may be because people with diabetes typically have high health care use, which could mean increased contact with the health care system (26). It may also be due to increased pressure from providers to quit.

The perceived risk of relapse due to weight gain was associated with lower odds of reporting tobacco abstinence, even after controlling for differences in incidence of depression. Findings from this study indicated that participants with diabetes were no more likely than the comparison group to gain weight over time. However, among participants who reported successfully quitting smoking, those with diabetes were more likely to report that they gained weight than were quitters who did not have diabetes. This weight gain may be attributable to the increased prevalence of obesity and depression in the diabetes arm. People with diabetes have been shown to have higher rates of depression (27), and depression and diabetes have both been associated with reduced self-care, including nonadherence to diet, exercise, and medication (28). Adding smoking cessation to the equation may further increase weight gain.

Although depression can impede successful quitting (29), those with diabetes were able to quit smoking at same level as the comparison group, despite having elevated levels of depression. These findings suggest that depression may not directly impact successful quitting in those with diabetes. However, since those with and without diabetes reported significantly different weight gain during prior quit attempts, the relationship between depression and quitting may be moderated by weight changes. Given that lower levels of weight concern were a significant predictor of successful quitting in this study, a full mediation and moderation analysis is warranted to clarify the relationship between depression, weight gain, weight concern, and quitting. When taken together, these weight-related findings suggest that greater efforts are needed to address weight gain in cessation treatment.

Numerous limitations should be considered when interpreting these results. First, results may not be generalizable to people outside of Washington State or to people who attempt to quit smoking through other treatment resources. Second, because the purpose of the study was to evaluate differences in use and quit rates, we matched by sex, insurance status, and cigarettes per day, thus limiting our ability to assess differences in these variables. Third, although only 6% to 10% of the initial sample refused to participate in the survey, the additional 26% to 33% who were located but not reached after 11 attempts might be considered passive refusals. Because of the low contact rate, it should also be noted that responder quit rates are tentative. Although the response rate for this study is lower than that of many clinical trials, it is comparable to completion rates from other telephone-based studies of quitline participants (12). Fourth, Medicaid fee-for-service data were not available for the entire study period. Although there is no reason to believe that the inclusion of additional Medicaid data would have changed these results, this is a limitation of the current study. Finally, diabetes diagnoses were self-reported and may underestimate the true population of quitline participants who have diabetes.

Despite these potential limitations, these data make an important contribution to the literature, suggesting that people with diabetes use the quitline and succeed at quitting at rates equal to those without diabetes, but that quit rates may be further enhanced by addressing weight concern and weight gain.
